# What makes an online problem-based group successful? A learning analytics study using social network analysis

**DOI:** 10.1186/s12909-020-01997-7

**Published:** 2020-03-18

**Authors:** Mohammed Saqr, Jalal Nouri, Henriikka Vartiainen, Jonna Malmberg

**Affiliations:** 1 University of Eastern Finland, School of Computing, Joensuu Campus, Yliopistokatu 2, fi-80100 Joensuu, Finland; 2 Department of Computer and System Sciences (DSV), Stockholm University, Borgarfjordsgatan 12, PO Box 7003, SE-164 07 Stockholm, Sweden; 3 University of Eastern Finland, School of Applied Educational Science and Teacher Education, Joensuu, Yliopistokatu 2, fi-80100 Joensuu, Finland; 4 Department of Educational Sciences and Teacher Education, Faculty of Education, University of Oulu, P.O. Box 2000, Oulu, Finland

**Keywords:** Learning analytics, Data analytics, Social network analysis, Social networking, Problem-based learning, Online learning, Small groups

## Abstract

**Background:**

Although there is a wealth of research focusing on PBL, most studies employ self-reports, surveys, and interviews as data collection methods and have an exclusive focus on students. There is little research that has studied interactivity in online PBL settings through the lens of Social Network Analysis (SNA) to explore both student and teacher factors that could help monitor and possibly proactively support PBL groups. This study adopts SNA to investigate how groups, tutors and individual student’s interactivity variables correlate with group performance and whether the interactivity variables could be used to predict group performance.

**Methods:**

We do so by analyzing 60 groups’ work in 12 courses in dental education (598 students). The interaction data were extracted from a Moodle-based online learning platform to construct the aggregate networks of each group. SNA variables were calculated at the group level, students’ level and tutor’s level. We then performed correlation tests and multiple regression analysis using SNA measures and performance data.

**Results:**

The findings demonstrate that certain interaction variables are indicative of a well-performing group; particularly the quantity of interactions, active and reciprocal interactions among students, and group cohesion measures (transitivity and reciprocity). A more dominating role for teachers may be a negative sign of group performance. Finally, a stepwise multiple regression test demonstrated that SNA centrality measures could be used to predict group performance. A significant equation was found, F (4, 55) = 49.1, *p* < 0.01, with an R2 of 0.76. Tutor Eigen centrality, user count, and centralization outdegree were all statistically significant and negative. However, reciprocity in the group was a positive predictor of group improvement.

**Conclusions:**

The findings of this study emphasized the importance of interactions, equal participation and inclusion of all group members, and reciprocity and group cohesion as predictors of a functioning group. Furthermore, SNA could be used to monitor online PBL groups, identify important quantitative data that helps predict and potentially support groups to function and co-regulate, which would improve the outcome of interacting groups in PBL. The information offered by SNA requires relatively little effort to analyze and could help educators get valuable insights about their groups and individual collaborators.

## Background

Problem-based learning (PBL) as an approach to instruction has attracted much attention across disciplines in higher education. PBL was first developed in medical education in the 1950s to respond to the criticism that traditional teaching methods fail to prepare medical students for solving problems in clinical settings [1]. After the successful development of PBL in various fields of medical education, it is now being implemented in higher education, professional education as well as in K–12 education [1, 2]. According to Hung, Jonassen, & Liu (2008), the rationale for PBL lies in its compatibility with modern educational goals such as the education of self-directed and life-long learners who can solve real-life problems in collaboration [1]. Nowadays, when researchers discuss twenty-first-century skills, they often emphasize key elements of PBL, such as being able to communicate and collaborate to solve complex problems, being able to adapt and innovate in response to new demands and changing circumstances, and being able to use technology to build new knowledge [3].

PBL originates on constructivist conceptions of learning by assuming that knowledge is constructed by the learners in their interactions with the environment [4, 5]. Since knowledge cannot be directly transmitted, PBL instruction should consist of learning activities and environments that facilitate knowledge construction [5]. The principal idea behind PBL is that the subject matter content and skills to be learned are organized around shared problems, rather than as a hierarchical list of topics delivered by the teacher [1]. In PBL, the students need to articulate the problem and then to search, evaluate, construct and share information, and apply it in the context of the problem-solving process at hand [6]. Accordingly, the ability to make insightful and productive use of the learning resources is playing an important part in PBL method [1, 6]. This is to say, PBL often challenges students, since it requires learners to engage in self-regulated learning [7], that includes, for example, ability to purposefully regulate own cognitive, motivational, and emotional behavior as well as that of others for optimal learning [8].

Collaborative learning is the second key element of PBL [9, 10]. By working together in small groups, students are expected to actively communicate, share their expertise and previous knowledge, make joint decisions, negotiate responsibilities, as well as to evaluate and modify the strategies of learning and group work through interactive dialogue [11]. As such, conceptualizing PBL and collaborative learning has also been influenced by sociocultural theory, which emphasizes the pursuit of a common goals through interpersonal interactions [12, 13]. From this perspective, individual group members represent interdependent self-regulating agents who at the same time constitute a social entity that creates affordances and constraints for group and individual learning [14].

Moreover, previous studies have shown that the success of collaboration relies on coordinating collaborative actions toward a shared goal [15, 16]. This is to say, in successful collaboration, each student needs to be in charge of their learning, coordinate their peer learning, and also coordinate the group learning as a whole [17]. This requires communication such as construction and pursuit of a shared object and collaborative planning [14, 18, 19], including search for common ground [16, 20], negotiation of roles and responsibilities [13], as well as distribution and organization of workload [13, 15]. Furthermore, collaborative learning requires equal team member participation that leads to advances and co-elaboration of the knowledge objects at hand [16, 21]. In other words, this type of interactional achievement is realized through shared epistemic agency, which emphasizes a capacity that enables individuals and groups to make appropriate judgments, to make plans and to coordinate the contributions toward joint outcome [16, 21]. Although there are substantial differences in conceptualizations of these intersubjective interactions and how these constructs are interpreted and defined, there seem to be an agreement that shared knowledge and joint cognitive responsibility are an essential aspect of coordinating and monitoring work in productive collaboration.

While research has shown that PBL can enhance, for example, interpersonal skills, communication skills and collaboration skills [22, 23], organization and dynamics of collaborative learning also impose many challenges for students and teachers as well [24]. Students may suffer from poor interaction and contribution of its members, the dominance of some members, poor time management, and focus on peripheral issues rather than the core of the problem under investigation [13, 14] If these challenges are not adequately addressed, they may cause student disengagement, task avoidance, dysfunctional group dynamics, and even withdrawal of one or more actors [13, 24, 25].

To summarize, there is a wealth of research on PBL that has helped understand many aspects and elements of collaborative learning. However, it is challenging to capture and support PBL with current instrumentation as they require time and efforts coding interactions, interviewing students, or surveying their responses. Furthermore, the current methods cannot be implemented to deliver automated analysis and insights for educators. Providing automated ways of analyzing and reporting about group functioning would help teachers offer better support for students, and focus on their teaching duties.

Accordingly, there is an evident need for methods, such as Social Network Analysis (SNA), that makes the collaborative aspects of the PBL process visible. SNA as a method provides automated monitoring and analysis of large volume of interactions, and requires little effort or manual intervention from educators. This study is trying to test the potentials of SNA in analyzing group functioning and to report which variables can help educators. The coverage of social network analysis will be introduced in the SNA section.

### Social network analysis

A social network is a group of connected entities through a relationship. Examples include a group of friends, collaborating companies, interacting union members countries, and extend to animal herds, proteins, genes, etc. Social network analysis is a group of methods that are used to study these connections. The entities are called “nodes”, “actors” or “vertices” and the relationships are called “links” or “edges”. SNA enables the study of how the relationships influence the behavior of the connected members, and how the structure shapes the embedded members. Proponents of the methods argue that the study of the structure and relationships could help understand learning, visually and quantitatively in a way not offered by the other qualitative methods [26].

Using SNA a researcher can map the patterns of interactions, highlight the active, the inactive and the isolated students. SNA could be also used to identify the roles of interacting students, for example, to identify the leaders, the moderators and the dominating participants. Furthermore, SNA analysis could be used to quantify interactions, positions of interacting actors, and the structural properties of their groups [27, 28]. This is usually done through the calculation of “importance” measures known as centrality scores, since importance may have different meanings in different situations, there are multiple centrality measures that quantify various perspectives. For example, indegree centrality is a measure of inbound interactions, closeness centrality is a measure of reachability by all collaborators. These measures have the potential to be used to describe collaborators’ behavior as well as their groups. Quantitative SNA may reveal important information also about the cohesion of groups, the flow of information and social capital. SNA could also offer a feasible and practical way of monitoring interactivity in collaborative settings. Furthermore, insights generated through automatic monitoring of interactions could serve as a basis for a learning analytics platform [29]. A further discussion of SNA metrics and interpretation will be presented in the methods section.

### SNA in healthcare education

SNA has become increasingly popular as a tool within the learning analytics ecosystem, however, studies of SNA in healthcare education are relatively limited compared to other methods [29]. Some examples exist, such as investigating the potentials of using SNA in PBL scenarios. For example, researchers have used SNA techniques to identify the roles of the students and tutors to get insights about gaps in the collaborative process, or use the centrality measures to predict performance using learning analytics methods [30–32]. While adding to our understanding of the process, these studies focused on the individual collaborator not the group as a unit of analysis. Some examples come from other domains such as [33], who created a recommender system based on group interactivity. Researchers used data from eight engineering courses and compared centrality measures to average group grades to recommend a better team composition [33]. Another example from Engineering used SNA to investigate the correlation between a team’s project score and the team balance; their results pointed to a positive correlation, although, the sample size was small [34].

This leads us to conclude that there is a gap in studies using quantitative methods, i.e. SNA and learning analytics to monitor, evaluate and provide insights for teachers who wish to provide support and feedback for their students. This study investigates the group as a unit through the lens of SNA and learning analytics. Our research focuses on the group dynamics, and how the elements of the PBL process influence the outcome of the group as a whole and the participating students.

The research questions of this study are as follows:

1. How do the groups, tutors and individual student’s interactivity variables of online PBL correlate with group performance?
2. Can the online PBL interactivity variables of the group be used to predict group performance?

## Methods

### Participants

Participants of the study were 598 participants (563 s-year dental students and 35 tutors). The courses were Body Health (teaches basics of physiology, pathology and disease of body health), Surgery (teaches foundations of surgery and common surgical principles), Neuroscience (teaches basics of nervous system physiology, histology pathology and common diseases), and Principles of Dental Sciences (teaches basic principles of dental sciences). The courses were repeated over three iterations in the year 2015, 2016, and 2017. Altogether, the participants worked in 60 groups (7 to 11 students and one tutor), mean = 10.6 students in each group.

### Context

The study was done in the Qassim University, College of Medicine in Saudi Arabia, which has adopted the PBL curriculum. The PBL tasks were open-ended scenarios, to provide a stimulus, for example a real-life clinical cases or problematic situations that are relevant in students’ future profession [1, 6]. The PBL process, which was organized similary for all courses, was structured as steps that occur on a weekly basis. Students are assigned randomly to small groups with a teacher on each course. On the first day of the week, students gather in a face-to-face meeting (2 h) where the group (with the teacher) has to clarify the terms, then they need to identify the problem, and formulate the learning objectives. Then the online stage takes place for the whole week. Working online in a threaded forum in the Moodle online learning management system, students are supposed to share their learning, debate, argue, refine together to reach a common understanding. At the end of the week the students synthesized and wrapped up their learning in another face-to-face session (2 h) [9]. At the end of the course, the students had an exam that measured their learning and understanding of the PBL topics. The university used the Moodle learning management system forum module and each PBL group had a separate online forum for each week. Online PBL was introduced to harness the potential of asynchronous collaborative learning that would give the student a platform for continuous interactions and engagement [35].

### Data

The data consists of Moodle logged written interactions from of 12 courses lasting for 7 to 8 weeks each. The interaction data were retrieved from the Moodle online system using custom database queries. The data retrieved includes the user ID, roles, grades, interactions and the metadata associated with each interaction (post author, group, course, thread, timing, subject, content, replies, and their metadata). Only interactions of the online PBL interactions were included. Posts related to announcements, course organization, or course questions were excluded. The terms user, actor or node are synonymous and therefore we will use the term user for simplicity throughout this paper.

### Data preparation

The collected interactions data were structured into a format compatible with the SNA analysis software the Igraph R! library [36]. To build the networks, edges were reconstructed from each post by considering the author of the post as the source and the replied-to-user as the target, and then all edges were aggregated into a full network for each group. The networks were *simplified,* so duplicate identical interactions between two nodes and self-replies were concatenated and were used as a weight for each edge. This approach preserves the network structure and interaction information while simplifying visualization and mathematical analysis.

### Data analysis and reporting

#### SNA analysis

There are three main components in the PBL process, the students, the tutors and the small group they are assigned. Therefore, the SNA analysis was done on these three levels: the individual user level (students and tutors) and on each group network level. The user-level analysis was done to calculate user activity metrics that reflect the quantity of interactions as well as the variables that reflect the user’s position and role in information exchange [37–40]. The variables that were calculated were as follows:

#### Individual-level variables

**Quantity of interactions metrics:** these metrics reflect how much a student has posted or replied and may be known as interaction analysis metrics.

**Outdegree:** Outdegree is the number of interactions by an user. It reflects the user’s effort, participation and social presence in the group [40–42].

**Indegree**^1^**:** Is the number of incoming replies or posts targeting a user. A user may receive a reply to an argument when it is worthy a reply; i.e. initiates a debate, opens a discussion or an argument. It is a reflection of the worthiness of a user’s interaction as evaluated by peers [37, 40, 41, 43].

**Closeness centrality:** Closeness centrality, is a measure of the distance from all others in the group, it represents the degree of connection and involvement in the discussion. Isolated actors tend to have low closeness centrality scores, and involved actors have higher scores. Two variants are calculated for directed networks, the closeness in for incoming interactions and closeness out for outgoing interactions [37, 40, 41].

**Betweenness centrality:** the number of times a user has connected participants, therefore, bridging them and relaying the information [40, 41].

**Eigen centrality:** measures the number as well as the strength of connections, a user connected to well-connected users would have higher scores [44].

**Hubness score:** based on the concept of Eigen centrality, however, it is directional and represents the value of connections to well-connected authorities and important users [44].

It is important here to mention that indegree and out-degree centralities were divided by the group number to account for variations in the number of group participants. All centrality measures including indegree and outdegree were scaled per course (from 0 to 1), to eliminate the course variations.

#### Group level variables

The small group acts as a networked system for information exchange. Thus the properties of the network of interactions may reveal important insights that may help teachers and learning designers. The calculated properties of each group represent group size, interactivity, cohesion and efficiency as a medium for interaction and information exchange. The calculated variables were the following:

**User count:** number of actors in each group.

**Edge count:** number of edges^1^ in each group.

**Mean distance** measures the reachability and ease of communication among group members, it is calculated by averaging the shortest paths among all group members. A higher distance indicates the presence of distant and isolated members of the group [38].

**Centralization:** centralization measures the distribution of a centrality score among actors in the same network. Therefore, indegree centralization is a measure of how incoming interactions are distributed among group members if a single user is receiving all interactions, the score is 1, a collaborative participatory network would have low scores. Similarly, outdegree centralization reflects the distribution of outgoing interactions in a group, centralization betweenness reflects the distribution of betweenness centrality, and so is centralization closeness [43].

**Transitivity**: the relative quantity of transitive friends, or how group members are connected through shared friends (forming a triangle), transitivity is a reflection of group cohesion [45].

**Reciprocity:** the ratio of interactions that are reciprocated. Interactions in collaborative participatory networks are reciprocated [45].

To further describe the group, we calculated the average centrality measures of individual members. Mean and median outdegree centralities represent the average quantity of interactions in the group. Mean and median indegree centralities represent the quantity of replies. Mean betweenness indicates the average brokering and mean closeness represents the average reachability of all group members.

### Performance

The performance of students was measured as the sum of the PBL grades. The PBL performance (20% of the grade) is assessed by the teacher according to student’s contribution to the PBL discussions each week. To increase uniformity, all groups are assessed by a single teacher each week. The second grade (80% of the grade) comes from the written exams (multiple choice and short essay questions) which tests the knowledge acquisition of the PBL objectives that were the subject of the interactions. Since grades comes from different courses, they were all standardized to eliminate the influence of course assessment variations. An ANOVA test was performed to test the difference among students’ mean grade in the included courses and showed that no statistically significant differences (ANOVA (F(11,551) = 0, *p* = 1)). The Levene’s test was performed to test for homogeneity of variance and demonstrated that the data did not violate the assumption of homogeneity.

Since there is no way to directly assess group performance, we used three measures

1. The mean normalized PBL grade of individual group members as a way of assessing the average grade of the group. Similar to [34].
2. The mean of each student’s difference between own grade (normalized per course) and normalized GPA of the past six courses before the year of the study to have a uniform baseline, to see if the student did better than their usual baseline.
3. Additionally, we included the fraction of improved students (number of improved students divided by number of students in the group) of the students who performed better than their GPA (normalized per course).

### Data analysis

SNA analysis was performed with Igraph library [36]. Each group data was considered a network, and were prepared for statistical analysis. The data were analyzed using R programming language [46]. Since many of the SNA variables did not follow a normal distribution, correlation tests were performed using Spearman rank correlation (Spearman’s rho). The R! library ‘StepReg’ was used to perform the Stepwise multiple linear regression [47]. Ten-folded cross-validation was performed to validate the model. The model was evaluated with Mean Squared Error (MSE), Root Mean Squared Error (RMSE), Mean Absolute Error (MAE) and R-squared. The level of significance for this study was set at 0.05.

## Results

### Descriptive statistics

In terms of online discussions of the group, the mean average distance was 1.51 (± 0.27), which is a short distance. In the case of online discussions, it could indicate that most students were close to reach and communicate. The mean reciprocity was 0.65 (± 0.22), indicating that most of the students replied to each other. The average indegree centralization was 0.27 (± 0.12), indicating that the replies were mostly distributed among students. However, mean outdegree centralization was 0.49 (± 0.27), indicating the presence of active members of some groups who participated more than others (hubs or leaders). Betweenness centralization was also quite low (0.22 ± 0.20). Table 1 lists the mean, median and standard deviation of group variables. The table also lists the 25th and 75th percentiles. Groups that has values below the 25th percentile have relatively low interactivity reflected as low quantity of interactions, low reciprocity and higher values of centralization indicating dominant actors.

**Table 1:** The network variables and characteristics of the interacting PBL group networks

Variable	Median	Mean	SD^a^	25th Percentile	75th Percentile
Edge count	177.500	214.3	164.318	85	274.250
Unique edges (simplified)	41.500	47.95	30.412	23	78
Mean degree	16.444	23.502	22.647	6.208	34.712
Mean distance	1.537	1.51	0.275	1.239	1.739
Transitivity	0.667	0.576	0.326	0.245	0.902
Reciprocity	0.690	0.651	0.216	0.522	0.817
Centralization in degree ^b^	0.249	0.266	0.121	0.174	0.341
Centralization out degree ^b^	0.453	0.488	0.265	0.220	0.7
Centralization Betweenness ^b^	0.131	0.225	0.193	0.047	0.325

^a^SD, ^b^Lower values are better

**Fig. 1:**
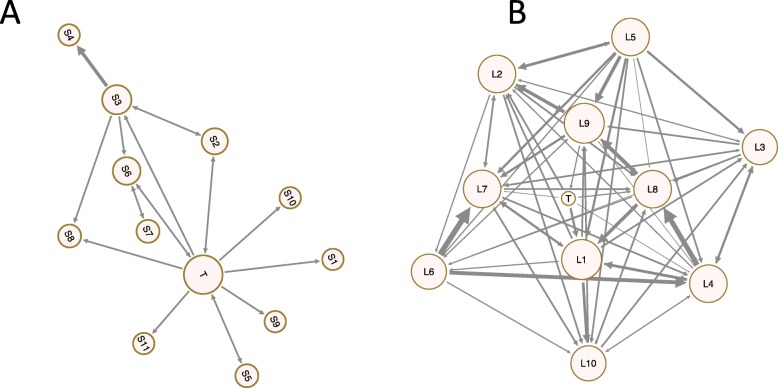
two groups from the dataset, the left group **a** is relatively inactive, centralized around the teacher with few interactions among students. Group **b** (right) is relatively active, teacher is involved but not dominant. Legend: Each circle represents a student, the circle with the letter T represents the teacher and every line represents an interaction, the circle size is proportional to the degree centrality and the thickness of the lines is proportional to the frequency of interactions

Figure 1 shows two groups of the dataset, group A is relatively inactive compared to group B, the Tutor – marked as T- is dominating, the visualization shows that the tutor in group A has more outgoing arrows (interactions) to most group members and so the tutor has high outdegree centralization. On the other side group B is interactive with interactions among most group members and the tutor. The SNA visualization is thus a powerful tool in showing how a group is doing and the relational aspects of interactions among group members. The mathematical or quantitative SNA analysis gives a more in-depth view of the roles in a quantitative way, that could help in a learning analytics context. We detail such analysis in the next sections.

### Correlation between group network properties and performance

Looking at how different properties of PBL group correlates with student’s performance we found that there was a significant negative correlation between the number of students in a group and the average normalized grade of the group (r (58) = − 0.29, *p* = 0.02), however, it was statistically insignificant when correlated with change in grade or fraction of improved students. This negative correlation should alarm course designers of the size of groups, as it may be a hindrance to learning.

The quantity of interactions^2^ in a group was significantly positively and moderately correlated with higher group’s grade, positive change in grades, and fraction of improved students.^3^ Furthermore, Group cohesion measures (transitivity and reciprocity) were all associated with better performance, positive change in grades and a higher ratio of improved students. That means that the quantity of students’ interactions^1^, replies to each other, and a broader base of participants of each group, is associated with a well-performing group and points to a functional group dynamic.

Contrary to signs of group cohesion, centralization measures were negatively correlated with the three performance measures (average group performance, change in performance or ratio of improved students). That was most obvious with centralization outdegree. An indication that when posting is concentrated or dominated by a few participants, it is not associated with better grades of the groups, or may be a sign of a dysfunctional group. Indegree centralization was also negatively correlated with lower average grades, but not as marked as the outdegree. Closeness and betweenness centralization (dominance of brokerage roles) were consistently negatively correlated with the three performance measures. In summary, an interactive cohesive group, that is not dominated by a member, and that have diverse participation among participants, is a group that is expected to score higher, have a positive change of performance and would help the higher ratio of students improve. These variables could be used as indicators for group function in a dashboard, that easily show the teachers the signs or red flags for a group that may need support. Table 2 has detailed statistics of all correlation results.

**Table 2:** Correlation between performance measures and SNA parameters of the group networks

Group variables	Grade		Difference in grade		Fraction of improved students	
	*r*	*p*	*r*	*p*	*r*	*p*
Number of students	−0.296	0.022	− 0.215	0.1	0.183	0.163
Edge count	0.506	< 0.001	0.478	< 0.001	0.557	< 0.001
Mean distance	−0.617	< 0.001	− 0.516	< 0.001	− 0.277	0.032
Centralization indegree	−0.267	0.04	−0.197	0.131	−0.068	0.604
Centralization outdegree	−0.681	< 0.001	−0.642	< 0.001	− 0.548	< 0.001
Centralization Betweenness	−0.56	< 0.001	− 0.489	< 0.001	−0.341	0.008
Centralization Closeness	−0.548	< 0.001	−0.537	< 0.001	− 0.437	< 0.001
Transitivity	0.665	< 0.001	0.576	< 0.001	0.511	< 0.001
Reciprocity	0.628	< 0.001	0.579	< 0.001	0.463	< 0.001

### Mean centrality measures of group members and the members’ performance

The results of the group’s average centrality measures (Table 3) confirm the previous findings reported in Table 2 that interactivity variables (average indegree and outdegree) were strongly and positively correlated with the group grade, and moderately with a change in group grade and fraction of improved students. Of notice is that the variance of outdegree as measured by standard deviation (indicating imbalance among participation) was strongly and negatively correlated with grade. The mean betweenness centrality of all groups was only positively significantly with the number of improved students; as for closeness centrality, it was consistently correlated with better group performance measures. In summary, interactivity is a good predictor of a group that will probably score high and helps more students perform better. These variables could also be used as indicators for group functioning.

**Table 3:** Correlation between performance measures and SNA variables of the group members

SNA variables	Grade		Difference in grade		Fraction of improved students	
	*r*	*p*	*r*	*p*	*r*	*p*
Mean indegree	0.609	< 0.001	0.552	< 0.001	0.564	< 0.001
Mean outdegree	0.609	< 0.001	0.552	< 0.001	0.564	< 0.001
SD^a^ indegree	0.113	0.391	0.156	0.235	0.322	0.012
SD outdegree	−0.625	< 0.001	−0.585	< 0.001	− 0.294	0.023
Median in degree	0.635	< 0.001	0.574	< 0.001	0.563	< 0.001
Median out degree	0.648	< 0.001	0.593	< 0.001	0.563	< 0.001
Mean betweenness centrality	−0.112	0.392	−0.034	0.795	0.272	0.035
Mean closeness in centrality	0.625	< 0.001	0.633	< 0.001	0.448	< 0.001
Mean closeness out centrality	0.554	< 0.001	0.58	< 0.001	0.416	0.001

^a^SD standard deviation

### Tutors and the group interactivity

Tutor’s quantity of interactions (indegree or degree centralities) were not correlated with any of the group performance measures, except for the negative correlation of tutor outdegree and number of students who improved. Being close or reachable to most students (high closeness centrality) was positively correlated with the performance of the group. However, the betweenness centrality of the tutors was negatively correlated with group performance measures. In the same vein the hubness score of the tutors was negatively correlated with the three performance measures. That is, students’ performance was negatively correlated when tutors were more central in the interactions. Tutors usually try to stimulate groups that are not active, and their activity is a sign of a dysfunction not a cause per se. We see these results as a sign of difficulty scaffolding online groups. Table 4 includes all the statistics in details.

**Table 4:** correlation between tutor centrality measures and performance measures

	Grade		Difference in grade		Fraction of improved students	
	r	p	r	p	r	p
InDegree	−0.060	0.669	−0.057	0.680	−0.171	0.216
Outdegree	−0.125	0.368	−0.203	0.141	−0.29	0.034
Closeness in	0.444	0.001	0.47	0.000	0.191	0.166
Closeness out	0.229	0.096	0.215	0.119	0.182	0.188
Betweenness	−0.451	0.001	−0.41	0.002	−0.264	0.054
Hubness score	−0.648	0.000	−0.598	0.000	−0.444	0.001

### Using PBL interactivity variables of the group to predict group performance

A Stepwise multiple linear regression test was performed using the interaction variables of the three elements of the PBL process (the student, the tutor and the group) to predict group grade (dependent variable) with 60 observations. A significant equation was found, F (4, 55) = 49.1, *p* < 0.01, with an R2 of 0.76. The estimate of the constant term was 2.394 with a standard error of 0.492. The explanatory variables that accounted for the variance in groups’ grades according to the last regression model were the Tutor Eigen centrality, the user count, and the centralization outdegree which were all statistically significant and negative. However, reciprocity in the group was a positive predictor of group improvement. In summary, a group where the tutor is connected to the less interactive students, with lower count of enrolled students, and a better distribution of interactions that are reciprocated is expected to perform better. Table 5 has the results of the regression model. The model did not violate collinearity statistics (Variance inflation factor). A 10-fold cross validation was performed to validate the model, the MSE was 0.144, the RMSE was 0.379, the MAE was 0.303 and the R2 was 0.733.

**Table 5:** Stepwise regression variables for predicting group grades using interaction variables

	Standardized Coefficients	
	Beta	Sig.
(Constant)		0.00
Tutor Eigen Centrality	−0.398	< 0.000
User count	−0.354	< 0.000
Reciprocity	0.204	0.013
Centralization outdegree	−0.255	0.014

## Discussion

In terms of group interactivity, the results of the study revealed structural patterns and group dynamics in PBL settings and the implications that they had on group performance. When taking a closer look at the network properties and performance, the analysis revealed that well-performing groups had more interactivity among the students. The interaction was also more equally distributed, indicating that the more active the students were replying to each other’s contributions, the better the learning outcomes were likely to be.

The results of the study also showed that the performance of the group clearly suffered if the interaction was disproportionately concentrated or dominated by a few participants. This implies that when one or more dominant members inhibit the participation of others, it may be a sign of a non-collaborative group that may not perform as expected. What is more, the analysis revealed that also the group size correlated with negative students’ performance. It may indicate that in a large group, equal participation and contribution in collaborative efforts is difficult to achieve.

What is more, students’ active participation, taking leader roles to foster peer collaboration was also evidenced to be more important than the tutor. Being close or reachable to most students (high closeness centrality) was positively correlated with performance of the group, while the tutor’s quantity of interactions was not correlated with the group’s performance variables. Additionally, the role of the tutor in information exchange by being a moderator (bridging the unconnected students), or a hub who leads the interactions, was significantly negatively correlated to the performance of the group. This means that when the tutor becomes dominating, it has a negative correlation with group performance, or be a sign of the tutor trying to help engage students by taking over the leader role.

Taken together, the results of this study indicate that active and reciprocal interactions between the students is particularly an important sign for successful group work in PBL settings. Active communication and equally distributed interaction may indicate that the group has found common ground that has been evidenced essential for collaborative learning [14, 19]. This finding is in line with earlier research in terms of advances of collaborative learning for individual learning suggesting that when learners collaborate, they generate strategies and problem representations that are unlikely to occur in individual learning [48] . Despite the effectiveness of collaborative learning, the group dynamics do not always function as expected [48], and thus, there is evident need for methodologies and tools that could help PBL groups to regulate their learning and joint work.

Methodologically, the present study sets an example of using SNA as a learning analytics tool for demonstrating network structures and group dynamics that predict the student performance and the factors that would help a group function. While previous studies have identified a number of variables which structures and influence the effectiveness of PBL [25, 49, 50], the current study takes one step further by showing how interactivity variables of PBL elements correlate with group performance with a large sample size. Although the absence of content analysis is a limitation of this study, the unique contributions of SNA is that it provides tools for automated analysis of large volume of interactions. Clearly, methodological progress made in automated content analysis will advance future research on PBL, however, it is still in the early stages.

Understanding of student interaction and participation also provides important information for adjusting tutor facilitation and course design in general. In practice, if analysis such as SNA would be further extended for temporal network analysis, such an analytical approach could be harnessed for educational contexts, so that, for example, instructors or tutors could immediately find out what types of interactions occur between the collaborating groups and then intervene if needed. Furthermore, this study has shown how certain quantitative interaction variables could be operationalized for supporting students to co-regulate their collaborative work. Such a tool could, for example, prompt PBL groups to increase awareness of unequal distribution of interactivity or low reciprocity, and thus, promote their metacognitive awareness. In similar vein, teachers could also use these insights for targeted intervention, and use the tool to evaluate the effects of the intervention for further adjustment and improvement [31]. Accordingly, there is a need to develop not only learning environments that support collaboration, but also advanced automated methods to capture group interactions [51]. At this moment, much of the research is going in that direction, but we are not there yet [52]. Therefore, we think that future research could capitalize on these findings and try to build tools that automate the analysis and reporting of students’ interactions, and test methods for timely support based on these insights.

## Conclusions

The findings of this study have demonstrated that quantitative accurate estimation of the role of students, tutors and the groups is feasible, easy to implement and notably useful. Variables reflecting the quantity of interactions, equal participation, inclusion of all group members, reciprocity and group cohesion were the predictors of a functioning group. Using these variables, social network analysis could be used to monitor and co-regulate online PBL groups, and potentially support the groups to function. The study has also demonstrated the possibilities and potentials of SNA techniques in PBL using a large sample of students and interactions, that could be used as a basis for automatic monitoring.

## Data Availability

The data contains students’ grades and private information and thus can be obtained through an application to the college ethical committee with appropriate procedure.

## Footnotes

1 Edge is basically a single post or an interaction as defined in detail in the data preparation section. We use the word interaction synonymously for simplicity.

2 Please note that the indegree and outdegree were weighted by group size and normalized.

3 Please note that all grades were normalized per course, and so the grades cosidered everwhere in the study are the normalized grades.
